# Modified Potato Starch as a Potential Retardant for Prolonged Release of Lidocaine Hydrochloride from Methylcellulose Hydrophilic Gel

**DOI:** 10.3390/pharmaceutics15020387

**Published:** 2023-01-23

**Authors:** Justyna Kobryń, Bartosz Raszewski, Tomasz Zięba, Witold Musiał

**Affiliations:** 1 Department and Chair of Physical Chemistry and Biophysics, Wrocław Medical University, Borowska 211A, 50-556 Wrocław, Poland; 2 Department of Food Storage and Technology, Faculty of Biotechnology and Food Science, Wroclaw University of Environmental and Life Sciences, Chełmońskiego 37, 51-630 Wrocław, Poland

**Keywords:** lidocaine hydrochloride, modified starch, release study

## Abstract

The problem of drug delivery often concentrates on the prolongation of drug activity. Application of natural polymers which are biodegradable and inexpensive is in the interest of many researchers. The aim of this study was the application of newly synthesized starch derivatives as potential functional excipients proposed for hydrophilic gel with lidocaine hydrochloride (LH) to prolong drug release from the hydrogel matrix. In our study, we investigated the effect of starch modified with citric acid on the release kinetics of LH using UV-VIS and Fourier-transform infrared spectroscopy (FTIR), differential scanning calorimetry (DSC), as well as viscosity and pH measurements. We demonstrated the effectiveness of citric-acid-modified starch in prolonging the release of LH from methylcellulose gel.

## 1. Introduction

**Figure 1:**
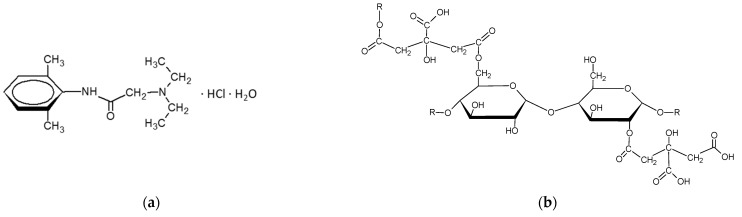
Structural patterns of lidocaine hydrochloride monohydrate (**a**) and of starch modified by citric acid cross-linking (**b**).

Lidocaine hydrochloride (LH) (Figure 1a) is a well-known anesthetic mainly used in dentistry, minor surgical procedures or to relieve pain during injections or disease conditions [1,2]. The external use of LH is widely available, and although allergic reactions to this drug are rare (1:1000) [3], it may cause discomfort, leading to discontinuation of the treatment. The introduction of drug derivatives such as articaine [4], or an attempt to administer LH in the form of a complex with a polymer [5], may be an alternative. The modern pharmaceutical science looks for innovative drug carriers in the field of newly synthesized or modified polymers [6,7]. One interesting group of polymers are the starch derivatives [8,9]. The native starch may be modified by several processes, including annealing [10], oxidation [11], cross-linking [12] and etherification [13]. The economically interesting and easily handled process is derivatization by esterification [14], developed, i.a., by Zięba [14,15] and Raszewski (Figure 1b).

Application of this sort of starch has been an interest of our group since 2020 [16], and was continued in [17]. The problem of drug delivery often concentrates on retardation of drug activity [18,19,20]. The main problem is proper evaluation of release kinetics, which may reflect the bioavailability of the drug, when applied orally or topically [16,18,21,22]. The LH is often applied topically when painful pathological symptoms occur [23]. The prolongation of the drug activity may beneficially influence the effects of application of the drug topically [22,24]. However, the interactions between the drug and the components of drug form may heavily influence the levels of the drug in the circulatory system [25]. In recent decades, studies of interactions between active substances and carriers have been based mainly on the analysis at the level of bonds between atoms in molecules: e.g., in studies of the molecule of LH which interacted with polymers (hydroxypropyl-β-cyclodextrin (HP-β-CD) and sodium saccharin) to mask the bitter taste of the drug [26]. In another study, the researchers prolonged the release of LH combined with the antibiotic amoxicillin trihydrate using a paste composed of Eudragit L 100 -55, carbopol 971 P, gum karaya powder and ethyl cellulose; the basic applied tools were Fourier-transform infrared spectroscopy (FTIR), differential scanning calorimetry (DSC) and UV-VIS spectrophotometry [27]. The interactions of LH complexed with β-cyclodextrin have been confirmed by UV-VIS, FTIR and thermogravimetry (TG) [5]. DSC analysis together with dielectric spectroscopy were used to study the interactions of LH with water molecules [28]. Determining the glass transition temperature using DSC was also necessary for evaluation of interactions between cassava starch and glycerol solution [29].

The aim of the work was the application of newly synthesized starch derivatives as potential functional excipients, proposed as components of hydrophilic gel with LH, for prolongation of drug release from this hydrogel.

## 2. Materials and Methods

### 2.1. Materials

The following materials were used in the study: methylcellulose (MC, Sigma-Aldrich CHEMIE, Steincheim, Germany), lidocaine hydrochloride monohydrate (LH, Sigma Aldrich CHEMIE, Steincheim, Germany) and native potato starch (NPS) (PPZ, Niechlów, Poland). Modified starch (S1–S4) was prepared by the University of Environmental and Life Sciences (Wrocław, Poland).

### 2.2. Methods

#### 2.2.1. Preparation of the Modified Starches

The process of cross-linking of NPS with citric acid was carried out with a slight modification of the roasting temperature, in accordance with the methodology developed by Klaushofer et al. (1978). Appropriate amounts of acid (2.5 and 5.0 g per 100 g dry weight of starch) were dissolved in 100 mL of water. The pH was adjusted to pH = 3.5 with 1 mol/L NaOH.

The obtained starch paste was left for 12 h at room temperature and then dried in an oven (Memmert, Germany) at 50 °C for 12 h. The samples prepared in this way were divided into two portions, which were roasted at two temperatures, 120 °C and 140 °C, respectively, for 3 h. Each sample was rinsed three times with 96% ethanol. The resulting precipitate was dried in an oven at 30 °C for 12 h, ground in a laboratory mill and sieved through a mesh of 400 µm [15]. The following starches S1–S4 were used in present study (Table 1).

#### 2.2.2. Preparation of Hydrophilic Formulations with Lidocaine Hydrochloride

Hydrogels based on MC were prepared according to Table 2. Two-thirds of the total formulation water was warmed up to 90 °C and MC was spread on the surface, mixed vigorously and cooled down. The LH was dissolved in the remained water at 25 °C, poured into the gel and left in the fridge at 4 °C for 24 h. The starch powder was added to the formed gels, mixed thoroughly and left for 12 h.

#### 2.2.3. Evaluation of the Release Kinetics of LH

##### Release Study

The release study was performed using cellulose membrane tubing of 14 kDa MWCO (Sigma-Aldrich, Saint Louis, MO, USA) as donor compartment immersed in the flask with 450 mL of distilled water as an acceptor compartment at a stirring rate maintained at 100 rpm. Two parallel measurements were performed in extraction cells by sampling 3 mL of the acceptor fluid with return of the medium at 25 °C every 5 and 10 min for 2 h. Analysis of the samples was performed using a UV/VIS spectrophotometer (Halo DB-20, Dynamica, UK) at 272 nm, according to the available bibliography [30]. The standard curve of three series of measurements with nine concentrations (0.01–0.666 mg/mL) enabled quantitative assay of LH.

##### Kinetic Models

The results were examined according to the zero-order kinetic, first-order kinetic, second-order kinetic and Higuchi models. The applied kinetic models are based on Fick’s first law, where the amount of substance diffusing in time through a given surface perpendicular to the direction of diffusion is proportional to the surface area, concentration gradient and time [31]. The zero-order model adheres to the case when the dissolved form of the drug does not aggregate, its surface does not change, and the equilibrium is not reached. This type of model usually reflects the sustained release process [32]. The first-order model shows the relationship between the dissolution of solid particles and surface interaction [31]. In the case of a second-order kinetics model, the process depends on both the units of time and the concentration of the drug. The Higuchi model is often used for the release of water-soluble and poorly water-soluble drugs contained in semi-solid and/or solid matrices [31]. The respective equations were gathered in Table 3.

#### 2.2.4. Differential Scanning Calorimetry (DSC) and Fourier-Transform Infrared Spectroscopy (FTIR) of LH Compositions with Modified Starches

LH was dissolved in distilled water, and appropriate amounts of S1–S4 were added to the solution, which is shown in Table 4. The components were mixed and left in a desiccator under vacuum for 72 h. After drying, the mixture was sieved through a mesh size of 1 × 1 mm. The dried suspensions were subjected to DSC and FTIR measurements.

#### 2.2.5. Fourier-Transform Infrared Spectroscopy (FTIR)

An infrared spectrophotometer with Fourier transformation (FTIR) and attenuated total reflectance (ATR) appetizer (Nicolet 380 FTIR, Thermo Scientific, Waltham, MA, USA) with OMNIC software was used to identify the possible interactions between LH and starch. The prepared formulations HS1–HS4 were compared with the standards of the pure substances. The spectra of powders were recorded at wavelengths of 400 cm^−1^ to 4000 cm^−1^ at 32 scans per sample and a resolution of 4 cm^−1^.

#### 2.2.6. Differential Scanning Calorimetry (DSC)

The samples of dried S1–S4, LH, their physical mixtures of 1:1 *w*/*w*, and dried formulations (HS1–HS4) were grated to a powder and studied using differential scanning calorimetry (DSC). The study was performed in differential scanning calorimeter (DSC 214 Polyma, Netzsch, Selb, Germany). Samples of 3–5 mg were scanned at a constant heating rate of 5 K/min in the temperature range from 0 to 350 °C, in aluminum containers under nitrogen flow of 50 mL/min.

#### 2.2.7. Determination of the Viscosity of the Formulations

The dynamic viscosity of formulations F1–F4, and REF was studied in the Höppler viscometer (Falling Ball Viscometer, Brookfield KF, Middleboro, MA, USA). The fall time of the steel ball in the gels at the angle of 70° was measured at 35 ± 0.5 °C and the viscosity was calculated according to the equation:

*μ* = *t*(*d*1 − *d*2)*KF* (1)

where *µ* is dynamic viscosity [mPa·s]; *t* is passage time of the ball [s]; *d*1 and *d*2 are densities of the ball and the samples, respectively [g/cm^3^]; *K* is the ball constant [mPa·cm^3^/g]; and *F* is a working angle constant.

#### 2.2.8. Determination of pH of Starch Samples and Evaluated Formulations

The pH of the formulations F1–F4 and REF was assessed in potentiometrically in CX601 device (ELMETRON, Zabrze, Poland) connected with electrode EPS-1 (ELMETRON, Zabrze, Poland) at 22.5 ± 0.5 °C.

#### 2.2.9. Visualization of the Starch Samples

The samples were visualized in a scanning electron microscope EVO LS15 (ZEISS, Oberkochen, Germany) at 20 kV after dehydration with ethyl alcohol and gold plating with Scancoat 6 sputtering machine (Edwards, London, UK).

## 3. Results

### 3.1. Evaluation of the Release Kinetics of LH from the Formulations

**Figure 2:**
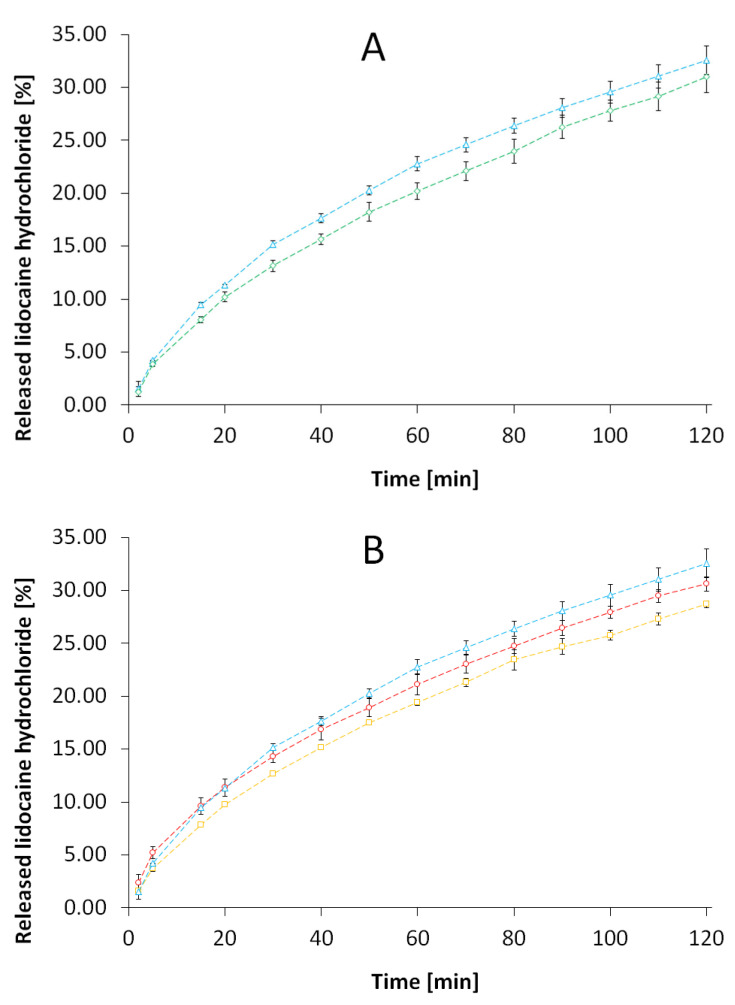

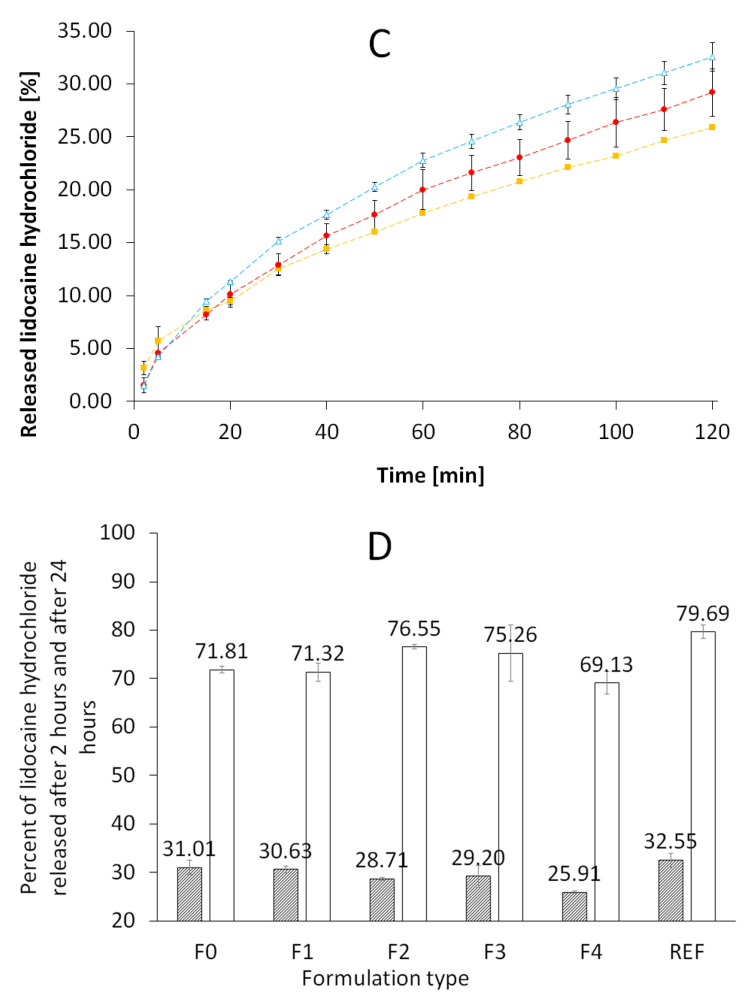
(**A**) The release course of LH from the formulation doped with low level of S1 (F0: -◊-, green markers). (**B**) The release course of LH from formulations doped with S1 and S2 (F1: -○-, red markers; F2: -▫-, yellow markers). (**C**) The release course of LH from formulations doped with S3 and S4 (F3: -●-, red markers; F4 -▪-, yellow markers). The plots on (**A**–**C**) are compared to methylcellulose gel (REF: -∆-, blue markers). (**D**) The percent of released LH after 2 h (shaded columns) and after 24 h (non-shaded columns). The abbreviations S1–S4, as well as F0–F4, REF are elucidated in Table 1 and Table 2, respectively. According to the Student’s *t*-test with Bonferroni correction, there is no statistical significance of the tested formulations in relation to the reference sample (α = 0.01).

The release courses of LH from the vehicles containing methylcellulose doped with the modified starches S1–S4 are presented in Figure 2. The amounts of released LH were highest in the case of methylcellulose gel, without added starch (formulation REF). Slight decrease of the released LH quantity was observed when small portion of S1 was added (Figure 2A). The released percentage of LH decreased with the increased percentage of citric acid in the starch added to the formulation, both in the case of starch roasted at a temperature of 120 °C (Figure 2B) and in the case of starch roasted at a temperature of 140 °C (Figure 2C). The Figure 2D gathers the final amounts of released LH after 24 h as a control. Respective statistical considerations were shortly given in Table S1. 

The parameters of kinetic equations of zero-order, first-order and second-order kinetics models as well as of the Higuchi model are presented in Table 5. The highest Pearson’s coefficients are in bold in Table 5.

### 3.2. Fourier-Transform Infrared Spectroscopy (FTIR)

**Figure 3:**
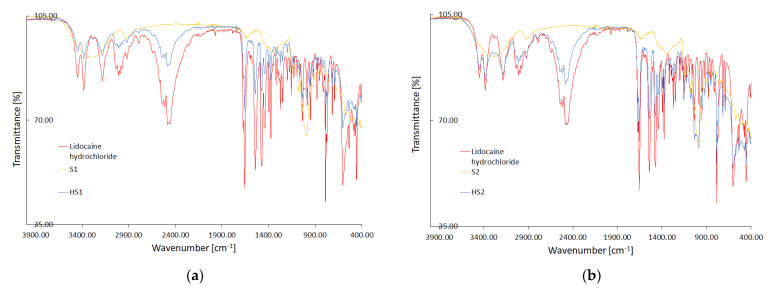

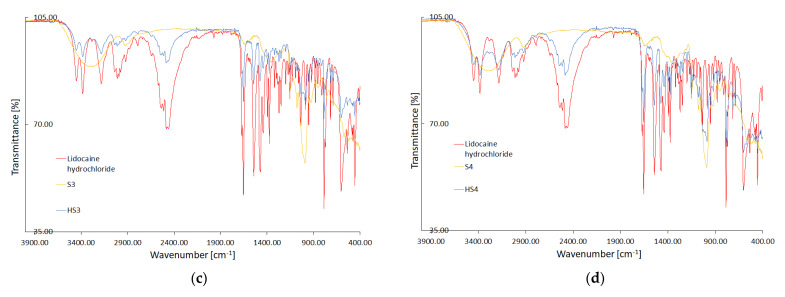
FTIR spectra of LH, modified starch (S1–S4) and experimental formulations of lidocaine hydrochloride with modified starch (HS1–HS4); (**a**–**d**) respectively. The abbreviations HS1–HS4 are elucidated in Table 4.

The spectra of pure LH, as well as the spectra of modified starches and formulations composed according to Table 4, are presented in Figure 3. On the graphs a–d, the plots of formulations HS1–HS4, were compared to respective modified starches S1–S4, and the FTIR spectrum of LH was attached in every case for detailed visualization.

### 3.3. Differential Scanning Calorimetry (DSC)

**Figure 4:**
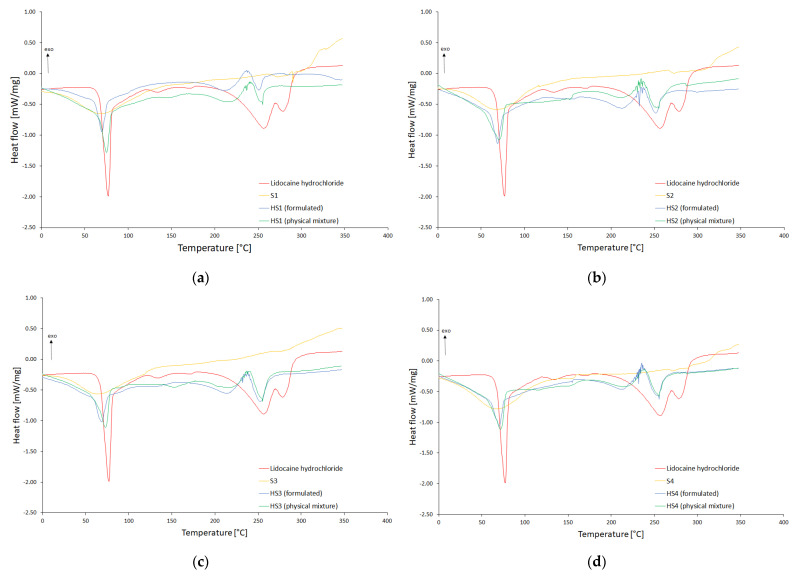

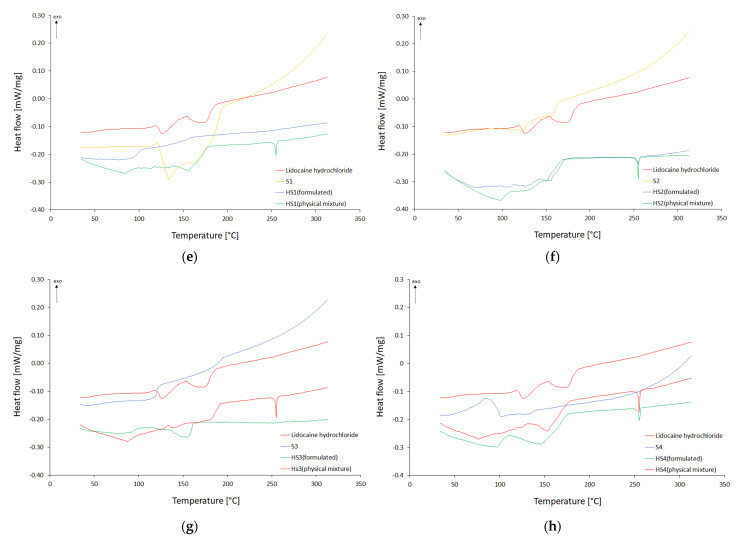
The DSC thermograms of lidocaine hydrochloride (LH); (**a**,**c**,**e**,**g**) thermograms of the first heating cycle; (**b**,**d**,**f**,**h**) thermograms of the second heating cycle, modified starch (S1–S4) and experimental formulations of LH with modified starch (HS1–HS4). The abbreviations HS1–HS4 are elucidated in Table 4.

The results of DSC analysis presented in Figure 4 include comparisons of pure lidocaine and modified starches thermograms to the physical mixtures of the components, as well as the to the formulations composed according to Table 4. The exothermic direction was assigned by the respective arrow (exo).

### 3.4. Determination of the Viscosity of the Formulations

**Figure 5:**
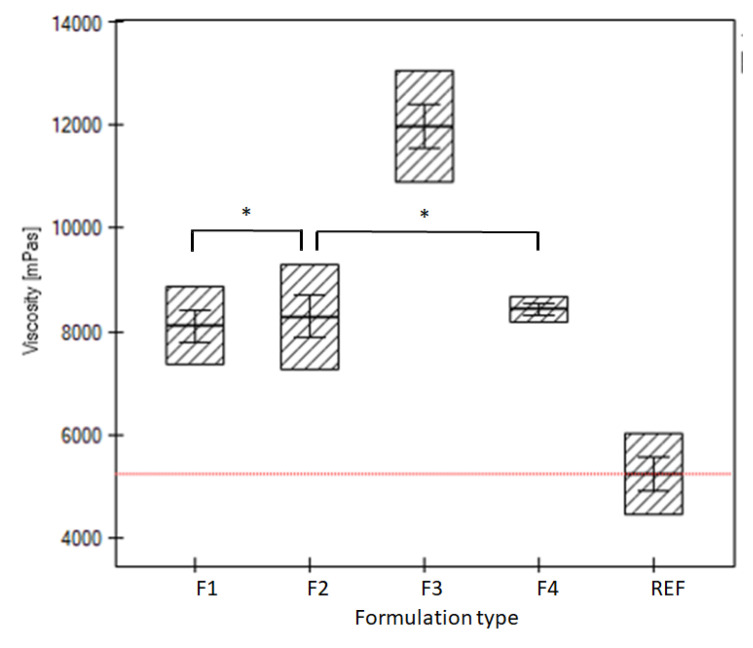
The viscosity of evaluated formulations F1–F4 and reference formulation REF (methylcellulose hydrogel). The red line refers to the mean viscosity of the REF. The abbreviations F0–F4 and REF are elucidated in the Table 2. An asterisk denotes the non-statistical significance of the formulation data according to the ANOVA statistical test for α = 0.05.

The viscosities of formulations F1–F4, and the reference formulation REF are gathered on the graph in Figure 5, with most pronounced viscosity in the case of formulation F3 and the lowest viscosity for reference gel REF. The statistical evaluation was presented in Table S2.

### 3.5. Determination of pH of Starch Samples and Evaluated Formulations

**Figure 6:**
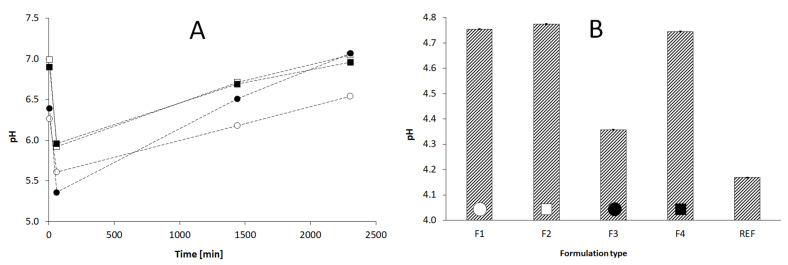
The pH of modified starch, S1: -○-; S2: -▫-; S3: -●-; S4: -▪- (**A**), applied in the respective evaluated formulations F1–F4 and reference formulation REF, and the pH of formulations F1–F4 and REF (**B**). The abbreviations S1–S4, as well as F1–F4 and REF are elucidated in the Table 1 and Table 2, respectively. The statistical significance of the formulation data observed according to the ANOVA statistical test for α = 0.05 (*p* < 0.05).

The pH of applied modified starches is presented in Figure 6A in the time perspective. The starch dispersed in purified water initially presented the pH in the range of 6–7. After 5 min the pH decreased to values between 5 and 6, and further increased up to the original values close to the range 6–7 after ca. 4 days. The Figure 6B presents the pH of hydrophilic gels with LH, where the F3 formulation as well as the REF formulation deviated out of the others (F1, F2 and F4).

### 3.6. Visualization of the Starch Samples

**Figure 7:**
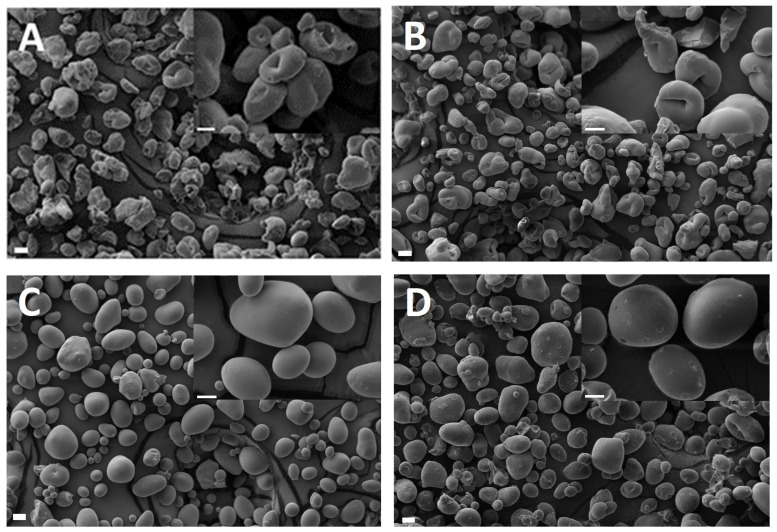
The SEM microphotographs of modified starch S1 (**A**), S3 (**B**), S2 (**C**) and S4 (**D**). The abbreviations S1–S4 are elucidated in the Table 1. The bar on the left on every microphotograph represents 20 µm, whereas on the enlargement (inserts right-above) the bar represents 10 µm.

The morphology of applied starch particles is presented in Figure 7A–D, with inserts, which enable more detailed insight on the surface of the particles.

## 4. Discussion

### 4.1. Evaluation of the Release Kinetics of LH from the Formulations

The initial parts of the release process are visibly curved on the graph, whereas the further parts are more straight-lined (Figure 2A–C). Thus, the initial stage of release should be further evaluated in the terms of more complex kinetic models, which may be applied in specific calculations. In any case, the increase of citric acid concentration in the starch prolongs the LH release in the first two hours, whereas after 24 h the results of other processes are observed, presumably hydrolysis of starch carrier.

The release patterns of evaluated samples of hydrophilic gel with LH gave variated results in the terms of kinetic model. The F0, F1, F4 and REF samples represented, according to the calculations, release model of Higuchi, which is usually applied to hydrophilic gels. In the case of F2, the second-order kinetics was indicated, whereas in the case of F3, the first-order kinetics. The present results are similar to our previous data which reflected, among others, β-escin release form methylcellulose gel with acetylated starches [16]. In the abovementioned research, the formulations of methylcellulose gel contained 20 to 40 percent of starch additive, and the resulting prevalent kinetic model was the second-order kinetics. However, the Higuchi model was identified in the case of methylcellulose gel without starch, so we suggested that the course of release depended on the concentration of the substance in the donor compartment. Moreover, there were two dynamic factors influencing the β-escin release. The Higuchi model describes the release of a drug from an insoluble matrix as the square root of a time-dependent process based on Fickian diffusion, as presented in Table 3. The Higuchi model was applied in preparations of tablets and microspheres by Gohel et al. [33]. According to their research, the spherical forms of carriers showed a better matching to this model compared to flat tablets, and the fit to the ideal model was best in the first 2 h of release. In this research, most of the samples, including reference, followed Higuchi release kinetics (Table 5). Presumably, the small amount of starch had a non-significant effect on the free flow of LH from the polymeric carrier into the solution present between the polymer branches, as may have been the case with the content of 20 or 40% (*w*/*w*) of the starch carrier in our earlier study [16]. This thesis is confirmed by fitting the starch-free methylcellulose gel to the Higuchi model. In addition, starch sphericity may have a greater effect on release retardation of LH than the content of functional groups derived from citric acid. This may confirm the highest slowdown of release in the case of S4 of highly spherical structure, both after 2 and 24 h. The formulation F4 showed the closest values to the statistical significance difference in relation to the reference gel REF in Student’s *t*-test with Bonferroni correction α = 0.01, *p* = 0.0130. Kanwar et al. [34] showed the influence of microcrystalline cellulose pellets sphericity on drug release and compared the release to the marketed tablets of prasugrel hydrochloride. The irregular and corrugated structure of S3 could also influence the release process, which was adapted to the first-order model [31]. However, the structure of the S3 starch particle alone is insufficient to alternate the release run pattern. In addition, the low pH could be a factor contributing to this change. The lower pH of the carrier could influence the ionization degree of LH and thus its release into the medium, as was detailed by Sjöberg et al. [35]. According to the Henderson–Hasselbalch equation, the low pH of the solution leads to a decrease in the number of non-ionized LH molecules, which may easily diffuse across the biological membrane [36]. The prospective area of research should include evaluation of the release process as a two-stage phenomenon. There is a substantial difference between the samples in the percentage of released LH after 2 h, compared to the respective controls after 24 h. The most probable elucidation is the difference in kinetic models which govern the evaluated processes. This hypothesis is supported by the fact that the regular pattern of prolongation with the increase of citric acid within the formulation was disturbed after 24 h (Figure 2D).

### 4.2. Fourier-Transform Infrared Spectroscopy (FTIR)

The FTIR spectrum of pure LH shows characteristic peaks of the N-H stretch bonds interacting at 3447 and 3382 cm^−1^, the C-H stretch bonds participating in the ring at 3180 cm^−1^ and a set of peaks from 3034 to 2920 cm^−1^ for the C-H bonds receiving in combination. Vibration at 1655 cm^−1^ indicates the presence of the C=O stretch bond, and peaks at 1540 and 1471 cm^−1^ are related to the C-C stretching bonds in the aromatic ring. The zone of peaks in the range 1000–1250 cm^−1^ reveals the C-N and the N-H interactions in the amides [26]. The starches’ FTIR spectra indicate a broad peak of the O-H stretching bond at about 3300 cm^−1^, the C-O stretching vibration at the range 1185–1347 cm^−1^ and the O-H bending bond at 988 cm^−1^ [37]. FTIR spectra of LH dissolved and mixed with starches do not show shifted peaks compared to pure lidocaine, but their intensity is significantly reduced, suggesting the existence of physical interactions between LH and starch [27].

### 4.3. Differential Scanning Calorimetry (DSC)

The DSC thermograms of LH in the first heating cycle presented endothermic peaks responsible for melting (77.29 °C) and the region of degradation of the molecule (134, 170, 257, 279 °C) (Figure 4a,c,e,g) [38]. The starch addition caused a shift and change in the amount of heat supplied to the system. The largest shift of the melting point peak to the left, i.e., a decrease in this temperature, compared to the physical mixture was observed for the formulation with S1, then S3 (Figure 4a,e). A smaller shift was observed in the LH starch formulation of S2 and no shift was observed at S4 (Figure 4c,g). These shifts and a decrease in the heat supply may indicate a decrease in crystallinity [39]. The addition of starch S1 to LH in the physical mixture (HS1 physical mixture) caused a change in the disintegration temperature (148, 216 and 255 °C), and for the dissolved LH system (HS1 formulated), the disintegration temperature was observed only at 210 and 247 °C. Starch S2 shifted the decomposition temperatures to 153, 213 and 254 °C for the HS2 physical mixture and 154, 213 and 252 °C for HS2 formulated. For the starch S3 in the physical mixture, the decomposition temperatures were 155, 216 and 255 °C, and for the dissolved LH formulation, they were 127, 214 and 255 °C. In the case of starch S4, the disintegration temperatures were 117, 149, 215 and 255 °C for HS4 physical mixture and 152, 211 and 255 °C for HS4 formulated. The thermograms in the second heating cycle in HS1 and HS3 formulated did not show any melting in the range of 200 to 300 °C (Figure 4b,f). While for HS2 and HS4 formulated melting peaks were visible (Figure 4d,h). That may suggest the formation of a stable system with starch roasted at 120 °C. The starch exerts a protective role and decreases the LH degradation. A similar situation of system stabilization was observed in the study of the eutectic mixture of and tetracaine by Gala U. et al. [38]. This may also indicate some interaction between the lidocaine, and S1 and S3, which may play a role in the prolongation of release of LH from the gels with modified starch.

### 4.4. Determination of the Viscosity of the Formulations

The high viscosity often favors the prolonged release of the active substance from the hydrophilic gel via increase of its intrinsic friction, which influences the diffusion constant. This pattern was well preserved for the period of 2 h when the LH was released, with exemption of F3 formulation, which had an extremely high viscosity of almost 12,000 mPas. For this formulation, the pH was also diminished compared to the other samples. It may be proposed that the high viscosity and low pH of F3 correlated with the level of citric acid and temperature of preparation process, however the resulting release was a compromise. The low pH (Figure 6B) could favor the solubility of lidocaine, whereas the high viscosity could result in increased intrinsic friction, so the effect on the release was balanced. The low pH of F3, comparable with the REF may be justified by comparison to the respective pH of additive S3. The preparation procedure of S3, as well as the level of citric acid could influence the recorded level of pH in F3.

The observed phenomenon, when the F3 deviated from other samples in the terms of pH and viscosity, may be affiliated with structure of the used starch types. S1 and S3 contain characteristic intussusceptions (Figure 7A,B). This suggests that the solvents present in the environment of the particles may penetrate and potentially absorb into the particles. Additionally, the adhesive properties may influence the increased viscosity for S3. The above supposed interplay in F3 may result in release similar to other formulations. Conversely, S2 and S4 are smooth and round (Figure 7C,D). Due to smooth and compact surface of S2 and S4, the penetration of solvents with solvates, including ions responsible for pH value, may be hindered. 

## 5. Conclusions

The temperature of roasting as well as the level of esterification factor influence the prolongation of LH release from methylcellulose hydrophilic gel. The starch roasted at 140 °C more effectively favors the prolongation of the release of the LH compared to the starch pregelatinized at 120 °C. The phenomenon is most likely related to the spherical structure of the particles roasted at 140 °C, compared to the flatter and more differentiated structure of the 120 °C roasted starch. The influence is clearly observed when compared to methylcellulose gel without starch additive. Additionally, the content of citric acid favors the release prolongation. Increase in the concentration of esterification factor, citric acid, results in prolongation of the release of LH, both in the case of starch roasted at 120 °C and 140 °C; however, the prolongation is more noticeable only in the case of starch modified in the temperature of 140 °C (S4). In addition, S4, due to the higher pH values compared to S3, results in the formation of higher number of non-ionized forms of LH. The concentrations required to obtain prolonging effect overcame the level of 2% of modified starch in 2% methylcellulose in the case of starch roasted at 120 °C with 2.5% of additive of citric acid. The release prolongation was observed in formulations with 3% of modified starch in 2.5% methylcellulose hydrophilic gel. The kinetic models gave inconsistent interpretation, presumably due to the two-stage process of release, which will be further studied. The most visible results are provided by DSC analyses, which indicate the most stable LH formulation in the case of S1 and S3, starches roasted in 120 °C.

## Figures and Tables

**Table 1:** Substrate composition and applied temperature during preparation of modified starches S1–S4.

Starch Type	CA, dm [%]	NPS, dm [%]	Roasting Temperature [°C]
S1	2.5	97.5	120
S2	2.5	97.5	140
S3	5.0	95.0	120
S4	5.0	95.0	140

CA—citric acid; NPS—native potato starch; dm—dry mass.

**Table 2:** Substrate compositions (C) of formulations (F) with modified starch and lidocaine hydrochloride in form of hydrophilic gels for release, viscosity and pH studies.

	C	LH [g]	MC [g]	S1 [g]	S2 [g]	S3 [g]	S4 [g]	Water [g]
F								
F0		2.0	2.0	1.0				95.0
F1		2.0	2.5	3.0				92.5
F2		2.0	2.5		3.0			92.5
F3		2.0	2.5			3.0		92.5
F4		2.0	2.5				3.0	92.5
REF		2.0	2.0					96.0

LH—lidocaine hydrochloride; MC—methylcellulose; the abbreviations S1–S4 are elucidated in Table 1.

**Table 3:** The applied kinetic equations.

Kinetic Process	Equation	Release Rate
Zero order	$Q_{t}=Q_{0}-K_{0}t$	$K_{0}=\frac{Q_{0}-Q_{t}}{t}\;$
1st order	$\ln(Q_{t})=\ln(Q_{0})-K_{1}t$	$K_{1}=\frac{1}{t}\ln\frac{Q_{0}}{Q_{t}}\;$
2nd order	$\frac{1}{Q_{t}}=\frac{1}{Q_{0}}+K_{2}t$	$K_{2}=\frac{Q_{0}-Q_{t}}{Q_{0}Q_{t}}\frac{1}{t}$
Higuchi model	$Q_{t}=Q_{0}-K_{H\;}t^{0.5}$	$K_{H}=\frac{Q_{0}-Q_{t}}{t^{0.5}}$

*K*_0_—zero order release rate; *K*_1_—first order release rate; *K*_2_—second order release rate; *K*_H_—Higuchi model release rate; *Q*_0_—initial percentage of the released drug; *Q*_t_—percentage of the drug released after time *t*.

**Table 4:** Substrate compositions (C) of formulations (F) of modified starch with lidocaine hydrochloride for FTIR and DSC measurements.

	C	LH [g]	S1 [g]	S2 [g]	S3 [g]	S4 [g]	Water [g]
F							
HS1		0.8278	1.6556				3.0
HS2		0.7949		1.5899			3.0
HS3		0.9725			1.9449		3.0
HS4		0.9790				1.9581	3.0

LH—lidocaine hydrochloride; MC—methylcellulose; the abbreviations S1–S4 are elucidated in Table 1.

**Table 5:** Release rates of lidocaine hydrochloride from assessed formulations of methylcellulose gel, doped with modified starch F0–F4, and from the reference formulation REF, calculated according to selected kinetic models from entire set of data—2 h.

Formulation	$K_{0}\;$	SD	$K_{1}\;$	SD	$K_{2}$	SD	$K_{H}$	SD
F0	2.37 × 10^−1^	6.80 × 10^−3^	2.89 × 10^−3^	1.05 × 10^−4^	3.54 × 10^−5^	1.57 × 10^−6^	3.11	0.08
r^2^	0.9692		0.9837		0.9933		**0.9984**	
F1	2.25 × 10^−1^	6.63 × 10^−4^	2.76 × 10^−3^	8.19 × 10^−6^	3.40 × 10^−5^	3.04 × 10^−7^	2.96	0.00
r^2^	0.9603		0.9767		0.9886		**0.9996**	
F2	4.09 × 10^−2^	4.33 × 10^−4^	9.65 × 10^−4^	3.24 × 10^−5^	2.64 × 10^−5^	1.75 × 10^−6^	1.91	0.02
r^2^	0.9376		0.9872		**0.9996**		0.9832	
F3	3.85 × 10^−2^	2.12 × 10^−4^	9.35 × 10^−4^	6.74 × 10^−6^	2.68 × 10^−5^	6.77 × 10^−7^	1.87	0.01
r^2^	0.9791		**0.9962**		0.9957		0.9957	
F4	1.87 × 10^−1^	7.12 × 10^−3^	2.20 × 10^−3^	7.94 × 10^−5^	2.59 × 10^−5^	8.93 × 10^−7^	2.35	0.09
r^2^	0.9679		0.9787		0.9871		**0.9964**	
REF	3.77 × 10^−2^	7.91 × 10^−4^	8.52 × 10^−4^	5.75 × 10^−5^	2.23 × 10^−5^	2.81 × 10^−6^	1.82	0.04
r^2^	0.9681		0.9908		0.9875		**0.9920**	

*K*_0_—zero order release rate; *K*_1_—first order release rate; *K*_2_—second order release rate; *K*_H_—Higuchi model release rate; r^2^—Pearson’s coefficient square. The abbreviations F0–F4 and REF are elucidated in Table 2.

## Data Availability

Not applicable.

## Supplementary Materials

The following supporting information can be downloaded at: www.mdpi.com/article/10.3390/pharmaceutics15020387/s1, Table S1: Comparison of the *p*-values of the tested samples and the reference sample based on the Student’s *t*-test with Benferroni correction (α = 0.01). The red values indicate non-statistical significance of the formulation data—released drug percent after 2 h (A) and 24 h (B); Table S2: Comparison of the p-values of the tested samples and the reference sample based on the ANOVA test (α = 0.05). The red values indicate non-statistical significance of the formulation data—viscosity.
