# Supplementary material for: Modified Potato Starch as a Potential Retardant for Prolonged Release of Lidocaine Hydrochloride from Methylcellulose Hydrophilic Gel
Source: Pharmaceutics. 2023 Jan 23;15(2):387. doi: 10.3390/pharmaceutics15020387 (PMC9966901; doi:10.3390/pharmaceutics15020387)
Supplement: Supplementary file 1 [file pharmaceutics-15-00387-s001.zip › pharmaceutics-2091801-supplementary.pdf]

pharmaceutics

Modified Potato Starch as a Potential Retardant for Prolonged Release of Lidocaine Hydrochloride from Methylcellulose Hydrophilic Gel

Justyna Kobryń [1], Bartosz Raszewski [2], Tomasz Zięba [2] and Witold Musiał [1,*]

[1] Department and Chair of Physical Chemistry and Biophysics, Wrocław Medical University, Borowska 211A, 50-556 Wrocław, Poland
[2] Department of Food Storage and Technology, Faculty of Biotechnology and Food Science, Wroclaw University of Environmental and Life Sciences, Chełmońskiego 37, 51-630 Wrocław, Poland
* Correspondence: witold.musial@umw.edu.pl

Supplementary materials:

Table S1. Comparison of the *p*-values of the tested samples and the reference sample based on the Student's *t*-test with Benferroni correction ($\alpha = 0.01$). The red values indicate non-statistical significance of the formulation data—released drug percent after 2 h (A) and 24 h (B).

**A: *p* value**	F0	F1	F2	F3	F4
REF	0.8808	0.5814	0.0570	0.3584	0.0130
**B: *p* value**	F0	F1	F2	F3	F4
REF	0.0861	0.1165	0.9153	0.8188	0.0747

Table S2. Comparison of the *p*-values of the tested samples and the reference sample based on the ANOVA test ($\alpha = 0.05$). The red values indicate non-statistical significance of the formulation data—viscosity.

***p* value**	F1	F2	F3	F4	REF
F1	-	0.156778	<0.000001	0.02012	<0.000001
F2	0.156778	-	<0.000001	0.220189	<0.000001
F3	<0.000001	<0.000001	-	<0.000001	<0.000001
F4	0.02012	0.220189	<0.000001	-	<0.000001
REF	<0.000001	<0.000001	<0.000001	<0.000001	-
